# Contribution of quantitative I-123-ioflupane SPECT/CT to the differential diagnosis of dementia with lewy bodies

**DOI:** 10.1007/s00259-025-07680-7

**Published:** 2025-11-27

**Authors:** George Karun Kurian, Paolo Salvioni Chiabotti, Caroline Hall, Noemie Lejay, Jérémy Perriraz, Olivier Rouaud, Adrien Depeursinge, Marie Nicod Lalonde, Niklaus Schaefer, Gilles Allali, John O. Prior, Mario Jreige

**Affiliations:** 1 Department of Nuclear Medicine and Molecular Imaging, Lausanne University Hospital and University of Lausanne, Lausanne, Switzerland; 2 Leenaards Memory Center, Department of Clinical Neurosciences, Lausanne University Hospital and University of Lausanne, Lausanne, Switzerland; 3 Institute of Informatics, School of Management, HES-SO Valais-Wallis University of Applied Sciences and Arts Western Switzerland, Sierre, Switzerland

**Keywords:** Dementia with lewy bodies, I-123-ioflupane, Dopamine transporter imaging, SPECT/CT, Quantitative imaging, Visual hallucinations, SUV (standardized uptake value)

## Abstract

**Purpose:**

Quantification of I-123-ioflupane uptake using modern SPECT/CT improves diagnostic accuracy for disorders affecting the nigrostriatal pathway. This study assessed whether absolute and relative standardized uptake values (SUV) could distinguish dementia with Lewy bodies (DLB) from non-DLB in patients with suspected DLB and explored associations with core clinical features, including visual hallucinations (VH) and REM sleep behavior disorder (RBD).

**Methods:**

Seventy-four patients (mean age 71.5 ± 9.26 years; 39% female) were retrospectively included. All underwent I-123-ioflupane SPECT/CT imaging with both iterative (Flash 3D) and quantitative reconstructions. SUVmax, SUVmean, and relative SUV (rSUV) values were extracted for the caudate, putamen, and striatum. Uptake metrics were compared between DLB and non-DLB groups and analyzed in relation to clinical features, including visual hallucinations (VH) and REM sleep behavior disorder (RBD).

**Results:**

Visual assessment classified 28/74 scans (38%) as abnormal (sensitivity 90%, specificity 80%, AUC 0.846). Quantitative SUVmax in the striatum and putamen showed the highest diagnostic performance (AUC up to 0.83). Striatal SUVmax remained an independent predictor of DLB in multivariable analysis (OR = 0.58, *p* = 0.003). Patients with VH had significantly lower striatal SUVmax than those without (*p* = 0.004), with an optimal cutoff of ≤ 6.0 g/mL (AUC = 0.70, sensitivity 64.3%, specificity 80.4%). No significant differences were observed for RBD.

**Conclusion:**

Quantitative I-123-ioflupane uptake assessment using SUV measures offers clinically relevant diagnostic value for differentiating DLB from other neurodegenerative diseases. It also helps in identifying patients with visual hallucinations, supporting the broader integration of SUV-based dopaminergic imaging into clinical workflows.

**Supplementary Information:**

The online version contains supplementary material available at 10.1007/s00259-025-07680-7.

## Introduction

 Dementia with Lewy bodies (DLB) is a common form of dementia that predominantly affects attentional, executive and visuospatial cognitive functions, with core clinical features including fluctuating cognition, recurrent visual hallucinations, and spontaneous Parkinsonism [1]. DLB accounts for 4–8% of all dementia cases, with an incidence of 3.5/100,000 person-years [2]. However, it is largely underdiagnosed, shown by the significant disparity between clinically diagnosed individuals and postmortem confirmation through neuropathology [3, 4]. DLB is difficult to differentiate from other clinical forms of dementia, and this distinction is paramount in order to avoid inappropriate use of neuroleptics [5]. Over the past decade, significant efforts have been made to improve the differential diagnosis of DLB, resulting in the publication of consensus clinical criteria [6, 7].

Functional imaging techniques, particularly dopaminergic single-photon emission computed tomography, have emerged as valuable tools for the diagnosis of DLB [8]. However, most studies to date have relied on visual interpretation or semi-quantitative approaches, such as striatal binding ratios (SBR), rather than absolute quantification. The literature on the use of standardized uptake values (SUV) derived from CT-guided or anatomically normalized reconstructions remains sparse, despite their potential to offer more objective and reproducible measures of striatal dopamine transporter loss [8]. This methodological gap is compounded by a lack of structured collaborations between memory clinics and nuclear medicine departments using harmonized diagnostic protocols and clinically adjudicated patient cohorts. Previous research and meta-analyses have shown that visual or semi-quantitative assessment using striatal binding ratios (SBR) remains the standard for diagnosing DLB [8–10]. More recently, multicentre data have proposed optimized SBR thresholds for differentiating DLB from non-DLB dementias [9], yet absolute quantification methods using standardized uptake values (SUV) remain scarcely investigated [11–13]. Semi-quantitative ratios are limited by inter-scanner variability and the use of arbitrary z-score thresholds [10], whereas absolute SUV quantification allows harmonized, reproducible measurement of tracer uptake across systems [11–13].

To address this gap, we evaluated the clinical utility of absolute and relative SUV quantification of I-123-ioflupane (DaTSCAN^®^; GE Healthcare, Chicago, IL, USA) using xSPECT reconstruction, in comparison to conventional visual interpretation. Specifically, we aimed to determine whether SUV-based metrics could reliably differentiate DLB from other neurodegenerative conditions presenting with overlapping clinical symptoms, and to identify optimal thresholds that could enhance diagnostic performance. In this context, we distinguish between the traditional semi-quantitative SBR, which expresses striatal binding relative to background counts, and the relative SUV (rSUV) used here, derived from fully quantitative reconstructions normalized to injected dose and body weight.

## Materials and methods

### Patient selection

**Fig. 1:**
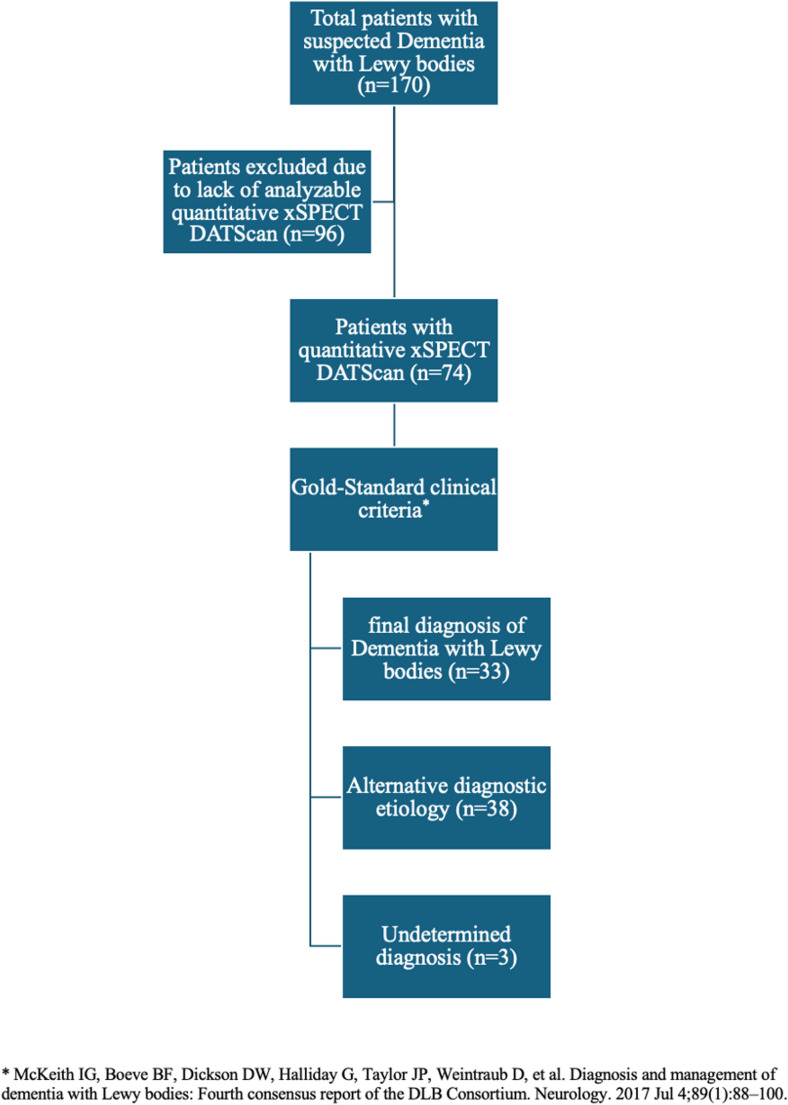
Flow chart showing the selection of patients for the retrospective analysis

A total of 170 patients were retrospectively screened from the cohort of the Leenaards Memory Clinic of the Lausanne University Hospital, based on referral for dopaminergic scintigraphic imaging (DaTSCAN) as part of a diagnostic workup for suspected DLB, in accordance with the most recent consensus criteria [6, 7]. Of the 170 screened patients, 96 were excluded because quantitative xSPECT data were unavailable or incomplete (non-xSPECT acquisitions, corrupted or missing raw data, incomplete CT attenuation correction, or severe motion artifacts). These predefined criteria were applied to ensure the reliability of SUV quantification and reflect real-world data completeness rather than selective exclusion. Additional exclusion criteria included age under 18 years and poor scan quality due to patient agitation or motor fluctuations. The final study population therefore comprised 74 patients with complete quantitative DaTSCAN SPECT/CT and clinical datasets (see Fig. 1). The mean (± standard deviation) age of the patients was 71.5 (± 9.2) years (range: 52.2–85.9), and 39% were female. Clinical collection of core DLB features (visual hallucinations, RBD, fluctuations) was available for 65/74 patients and used in subgroup and multivariable analyses where applicable.

Final clinical diagnoses were established by a multidisciplinary team comprising a neurologist, neuropsychologist, and nuclear medicine physician, in accordance with the 2017 consensus criteria for dementia with Lewy bodies. Diagnoses were confirmed after longitudinal clinical follow-up between June 2017 and June 2024, based on comprehensive review of all available clinical, neuropsychological, and imaging information from the patients’ electronic medical records at the memory clinic. This multidisciplinary reassessment minimized potential diagnostic circularity and strengthened the reliability of final classifications, acknowledging the possibility of underreporting of some core clinical features, such as visual hallucinations, in cognitively impaired patients. For non-DLB cases, the alternative etiologies included Alzheimer’s disease, Parkinson’s disease with dementia (PDD), frontotemporal dementia (FTD), corticobasal degeneration (CBD), vascular dementia, progressive supranuclear palsy (PSP), multisystemic atrophy (MSA), as well as secondary and rarer causes such as normal pressure hydrocephalus (NPH), neurodevelopmental conditions, mood disorders, alcohol-related or hypoxic brain injury, iatrogenic causes, and functional neurological disorders. Demographic and clinical characteristics of the study population are summarized in Table 1.

**Table 1:** Summary of patient characteristics

Variable	Non-DLB (*n* = 41)	DLB (*n* = 33)	*p*-value
Age (mean, years)	71.4 ± 9.6	71.8 ± 8.8	0.867
Male Gender (%)	20 (51%)	25 (76%)	0.05
Fluctuations (%)	11 (26%)	19 (58%)	0.015
Parkinsonism (%)	23 (59%)	26 (79%)	0.043
Visual Hallucinations (%)	5 (12%)	11 (33%)	0.041
RBD (%)	4 (10%)	9 (27%)	0.06

The local Ethics Research Committee of the State of Vaud approved the research protocol (CER-VD #2018 − 01513). Considering the retrospective nature of this study, the need for obtaining informed consent from the patients was waived.

### DaTSCAN acquisition and analysis

All patients underwent dedicated brain SPECT/CT acquisition using low-energy high-resolution (LEHR) collimators on a Siemens Symbia Intevo system (Erlangen, Germany). Imaging was centered on the striatum and basal ganglia, using a continuous 360° rotation with 60 projections acquired for 30 s each in a 256 × 256 matrix. The mean injected activity was 179 ± 17 MBq of I-123-ioflupane, and data were acquired on average 4 h 42 min post-injection. A low-dose CT scan (fixed 110 kV, 12 mAs) was performed for attenuation correction. Images were reconstructed using conventional iterative reconstruction (Flash 3D) and quantitative xSPECT. Flash3D (non-quantitative iterative reconstruction) was used for clinical visual assessment, whereas quantitative xSPECT was used for SUV extraction. Both pipelines incorporated CT-based attenuation correction, vendor scatter correction, and resolution recovery, using fixed vendor-recommended parameters kept constant across all patients. Quantitative xSPECT enabled SUVbw computation from system-calibrated data. SUVbw values are expressed in g/mL, consistent with DICOM quantitative imaging conventions, as they represent activity concentration normalized to injected dose and body weight. Our SPECT/CT systems undergo routine quantitative calibration and performance verification under the supervision of a certified medical physicist as part of clinical quality assurance, ensuring consistency of xSPECT-derived SUV metrics across acquisitions. All studies were visually inspected for patient motion prior to reconstruction; scans with non-correctable motion were excluded. When feasible based on patient habitus, the radius of rotation was kept below 15 cm to optimize spatial resolution. Medication lists were reviewed to identify agents known to interfere with I-123-ioflupane binding, and imaging was scheduled accordingly when modifications were clinically indicated.

The complete acquisition, including low-dose CT and reconstruction, required approximately 30 min. Quantitative reconstruction required approximately 5–7 min per study, adding only a few minutes compared with standard clinical reconstructions and remaining feasible for routine practice, particularly when automated processing is available. Visual interpretation was retrospectively retrieved from standardized clinical reports prepared by a junior nuclear medicine physician and reviewed by a senior physician, in accordance with EANM/SNMMI guidelines for assessing striatal symmetry and intensity. Each scan was categorized as normal or abnormal based on striatal uptake patterns, and final classifications reflected consensus between both readers. Data were analyzed using MI Neurology in *syngo*.via (Siemens Healthineers) by a co-registration of the individual tomographic data to a reference template followed by the application of a standard set of volumes of interest (VOI) and fully-automated extraction of mean regional counts and SUV values for the left and right caudate nucleus, putamen and striatum, as well as for the occipital region, taken as reference region for the calculation of relative SUV (rSUV). A representative example of the standard VOIs used for quantitative analysis, including the caudate nucleus, putamen, striatum, and occipital reference region, is shown in supplementary Fig. 1. In parallel, semi-quantitative analysis based on visual interpretation and standard striatal binding ratio (SBR) assessment was retrieved from clinical reports to allow comparison with the quantitative SUV-based approach.

### Statistical analysis

Continuous variables were reported as mean ± standard deviation (SD) and range. Categorical data were analyzed using Fisher’s exact test or the chi-squared test, as appropriate. For comparison of two groups (DLB vs. non-DLB), Student’s t-test was used when assumptions for parametric testing were met; otherwise, the Mann–Whitney U test was applied.

Quantitative I-123-ioflupane uptake metrics (SUVmax, SUVmean, and relative SUV [rSUV]) were compared between clinically diagnosed DLB and non-DLB patients for each region of interest (striatum, putamen, and caudate nucleus). Receiver operating characteristic (ROC) curve analysis was performed to assess diagnostic performance, with optimal cut-offs determined using the Youden index. Area under the curve (AUC), sensitivity, and specificity were reported.

Effect sizes were calculated using Cohen’s d to quantify the magnitude of differences in SUV metrics between DLB and non-DLB groups.

Multivariable logistic regression was performed to assess the independent association between quantitative SUV metrics and the final clinical diagnosis of DLB, adjusting for age, sex, and core clinical features including parkinsonism, visual hallucinations, REM sleep behavior disorder (RBD), and cognitive or motor fluctuations. Results are reported as odds ratios (OR) with corresponding 95% confidence intervals. Predicted probabilities were also computed from the model to enhance clinical interpretability.

Statistical analysis was performed using STATA (version 18.0; StataCorp, College Station, TX, USA) and Python (version 3.11, with scikit-learn and statsmodels libraries). Statistical significance was set at *p* < 0.05.

## Results

### Study population

A total of 74 patients were analyzed. Visual interpretation results were derived from FLASH3D reconstructions, whereas quantitative metrics (SUVmax, SUVmean, and rSUV) were extracted from xSPECT reconstructions. Based on final clinical diagnostic criteria used as the reference standard, 33 of the 74 patients (45%) were diagnosed with DLB. Among patients with DLB, 58% exhibited cognitive and/or motor fluctuations, 79% had parkinsonism, 33% experienced visual hallucinations, and 27% presented with REM sleep behaviour disorder (RBD), either at the time of clinical work-up or based on medical history. In all cases except 3, a final diagnostic aetiology was found for the DLB mimics, including Alzheimer’s disease (AD) (*n* = 9), Parkinson’s disease with dementia (PDD) (*n* = 1), frontotemporal dementia (DFT) (*n* = 1), corticobasal degeneration (CBD) (*n* = 1), normal pressure hydrocephalus (NPH) (*n* = 2), vascular dementia (*n* = 13), mood disorders (*n* = 7), and other causes such as psychotic disorders (*n* = 1), epilepsy (*n* = 1), and iatrogenic (*n* = 2).

### Diagnostic performance of quantitative I-123-ioflupane metrics

**Fig. 2:**
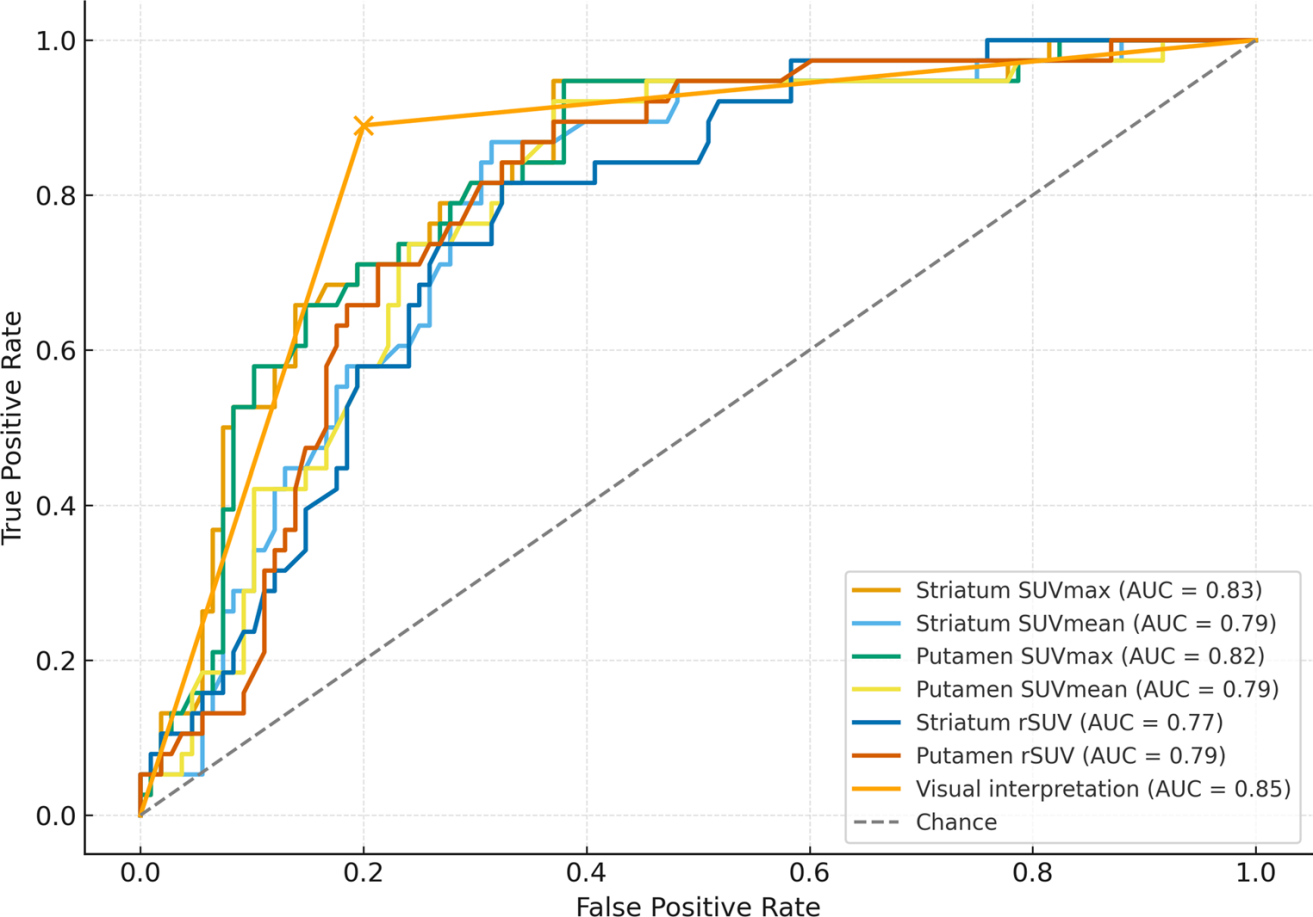
Receiver operating characteristic (ROC) curves for absolute SUV and relative SUV (rSUV) metrics derived from quantitative xSPECT, with the visual interpretation curve overlaid (AUC = 0.85).Each curve reflects the diagnostic accuracy of the respective metric, with area under the curve (AUC) values provided in the legend

Diagnostic performance metrics are summarized in Table 2 and illustrated in Fig. 2. Based on visual assessment and relative uptake metrics, 28 out of 74 (38%) scans were visually classified as abnormal. When using the final clinical diagnosis of DLB (*n* = 33) as the reference standard, the visual interpretation of I-123-ioflupane scintigraphy showed a sensitivity of 90%, specificity of 80%, and an area under the ROC curve (AUC) of 0.846.

**Table 2:** Diagnostic performance of quantitative SUV metrics for the final clinical diagnosis of dementia with lewy bodies (DLB). Receiver operating characteristic (ROC) analyses were performed for each region and metric. Optimal cut-offs were determined using the Youden Index. Both absolute (SUVmax and SUVmean) and relative (rSUV) uptake values are reported. AUC = area under the curve

Region	Metric	AUC	Cutoff	Sensitivity	Specificity
Striatum	SUVmax	0.828	7.21	0.947	0.63
	SUVmean	0.786	3.04	0.868	0.685
	rSUV	0.77	2.11	0.816	0.676
Putamen	SUVmax	0.823	7.21	0.947	0.62
	SUVmean	0.789	3.87	0.921	0.63
	rSUV	0.794	2.63	0.868	0.657
Caudate	SUVmax	0.798	5.35	0.737	0.778
	SUVmean	0.68	2.35	0.711	0.62
	rSUV	0.688	1.6	0.763	0.63

Quantitative analysis of absolute standardized uptake values (SUV) in the putamen, striatum, and caudate nucleus revealed significant differences between DLB (*n* = 33) and non-DLB (*n* = 41) patients. Absolute SUVmax, SUVmean, and rSUV significantly differentiated DLB from non-DLB in all three regions: caudate (*p* < 0.005, *p* = 0.028, *p* < 0.005), putamen (*p* < 0.005 for all metrics), and striatum (*p* < 0.005 for all metrics).

ROC curve analysis was performed using the final clinical diagnosis of DLB (*n* = 33) as the reference standard. Among the tested absolute uptake metrics, SUVmax of the striatum and putamen yielded the highest diagnostic performance. The area under the curve (AUC) for striatum SUVmax was 0.83, with an optimal cutoff of 7.21 g/mL (sensitivity 95%, specificity 63%). Similarly, putamen SUVmax had an AUC of 0.82 with the same threshold (7.21 g/mL), achieving 94.7% sensitivity and 62.0% specificity. The caudate SUVmax yielded an AUC of 0.80 with a cutoff of 5.35 g/mL, sensitivity of 74%, and specificity of 78%. SUVmean values from the same regions showed slightly lower diagnostic performance (AUC 0.68–0.79) but still retained clinically useful sensitivity and specificity profiles.

In addition, we evaluated relative SUV (rSUV), defined as the ratio of regional uptake to the occipital reference region. The putamen rSUV achieved an AUC of 0.79 with an optimal threshold of 2.63, followed by the striatum rSUV (AUC = 0.77, cutoff 2.11), and the caudate rSUV (AUC = 0.69, cutoff 1.60). These findings suggest that both absolute and relative quantification methods provide strong discriminatory power for identifying dopaminergic degeneration consistent with DLB. All results are summarized in Table 2 and visualized in Fig. 2 (see ROC curves).

**Fig. 3:**
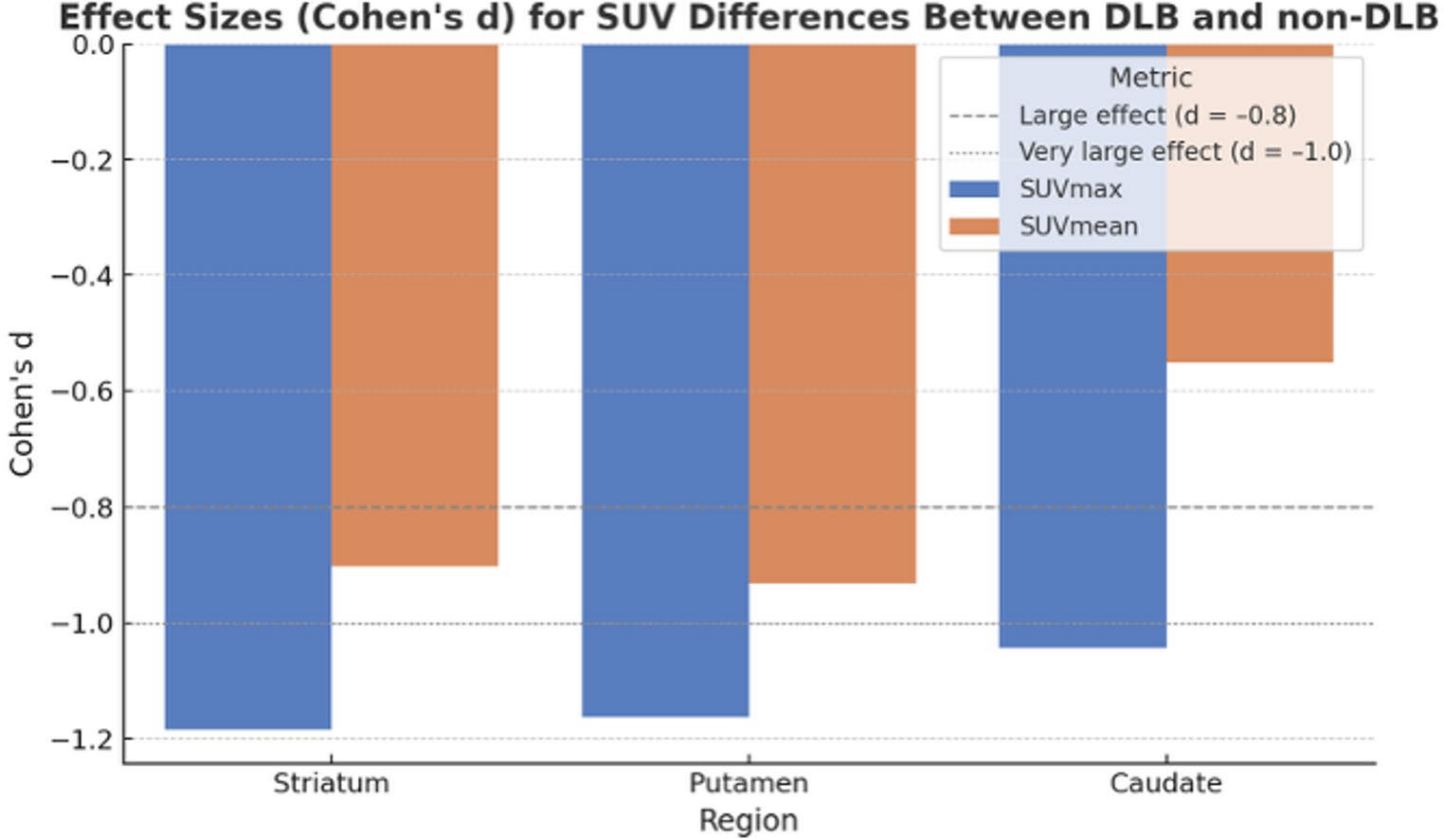
Cohen’s d effect sizes for SUVmax and SUVmean in the striatum, putamen, and caudate nucleus. All metrics show large effect sizes (d < − 0.8), indicating strong dopaminergic differences between DLB and non-DLB patients. Dashed and dotted lines mark the conventional thresholds for large (–0.8) and very large (–1.0) effects

Effect size analyses showed large group differences in SUV values between DLB and non-DLB patients. Cohen’s d values ranged from 0.90 to 1.18 for SUVmax and SUVmean metrics in the striatum and putamen, indicating strong separation of uptake distributions. These findings complement the ROC and logistic regression results and confirm the clinical relevance of the observed quantitative differences (Fig. 3).

### Multivariable and univariate predictors of clinical DLB diagnosis

In multivariable logistic regression, SUVmax of the striatum remained a significant independent predictor of DLB diagnosis (OR = 0.58, 95% CI: 0.41–0.83, *p* = 0.003), even after adjustment for age, sex, and core clinical features. While female sex and the presence of visual hallucinations showed trends toward association with DLB (OR = 0.33 and 2.92, respectively), these did not reach statistical significance. Age and parkinsonism were also not significant predictors in the multivariable model. (Table 3)

**Table 3:** Multivariable logistic regression analysis of predictors for the final clinical diagnosis of dementia with lewy bodies (DLB). Odds ratios (OR), 95% confidence intervals (CI), and p-values are reported for each predictor. Striatal SUVmax was independently associated with DLB diagnosis after adjusting for age, sex, and clinical features

Predictor	Odds Ratio (OR)	95% Ci Lower	95% Ci Upper	*P*-Value
SUVmax striatum	0.58	0.41	0.83	0.003
Age	0.97	0.91	1.05	0.473
Sex	0.33	0.07	1.49	0.151
VH	2.92	0.62	13.83	0.176
RBD	1.74	0.31	9.58	0.526
Fluctuations	1.3	0.27	6.13	0.744
Parkinsonism	0.86	0.17	4.24	0.848

In univariate analyses using Fisher’s exact test, the clinical features of visual hallucinations (*p* = 0.018), RBD (*p* = 0.013), and fluctuations (*p* = 0.002) were each significantly associated with the final diagnosis of DLB. These features may share predictive overlap with dopaminergic dysfunction captured by SUV quantification and were modeled with caution due to collinearity and sample size limitations.

### Quantitative differences in I-123-ioflupane uptake by clinical features

Clinical data on visual hallucinations (VH) and REM sleep behavior disorder (RBD) were available in 70 patients. Among them, 16 patients (22.9%) had VH and 13 (18.6%) had RBD. Striatal SUVmax values were significantly lower in patients with VH compared to those without (*p* = 0.004). In contrast, SUVmax values did not significantly differ between patients with and without RBD (*p* = 0.087).

ROC analysis for VH prediction identified an optimal SUVmax threshold of < 6.00 g/mL in the striatum, yielding a sensitivity of 64%, specificity of 80%, and an AUC of 0.70. This supports the potential value of dopaminergic quantification for identifying VH in the context of suspected Lewy body disease.

## Discussion

Our study demonstrates that quantitative assessment of I-123-ioflupane uptake using xSPECT reconstruction effectively distinguishes patients with DLB from non-DLB mimics. In a cohort of 74 clinically adjudicated cases, quantitative SUV metrics—particularly striatal and putaminal SUVmax—were significantly lower in DLB, yielding strong diagnostic performance (AUC up to 0.83). Striatal SUVmax achieved excellent sensitivity (95%) but moderate specificity (63%). This pattern reflects the high sensitivity of quantitative DaTSCAN imaging for presynaptic dopaminergic deficits, while mild reductions in other neurodegenerative or parkinsonian syndromes may lower specificity. Although higher specificity would be desirable for definitive diagnostic confirmation, this trade-off remains clinically acceptable for confirmatory imaging where ruling out DLB in uncertain cases is important. Further harmonization of SUV thresholds across scanners may enhance specificity.

Although visual interpretation achieved slightly higher AUC values (AUC = 0.85), quantitative SUV analysis provides objective and reproducible metrics that complement visual reads, particularly in borderline or early-stage cases where visual assessment alone may be equivocal. Both absolute and relative SUV measures provided robust discrimination, and SUVmax of the striatum emerged as an independent predictor of DLB in multivariable analysis, even after adjustment for age, sex, and core clinical features.

Our results align with those of Kuo et al. [9], who reported optimal DaTQUANT SBR thresholds for differentiating DLB and Parkinson’s disease, with an AUC of approximately 0.84. While their analysis relied on semi-quantitative ratios, our CT-based absolute SUV quantification achieved comparable diagnostic accuracy (AUC = 0.83), underscoring the methodological complementarity. Additional evidence supports early degeneration of the nigrostriatal dopaminergic pathway in both DLB and PD, as shown by multimodal imaging studies combining DAT binding ratios with MRI [13]. Similarly, Sato et al. [11] compared quantitative indices across dopaminergic (DLB and PD) and non-dopaminergic disorders (essential tremor, drug-induced parkinsonism, AD, and depression). They reported that both DVRs (distribution volume ratio) and SBRs performed similarly to SUV values, although SUVmax and SUVmean achieved the highest diagnostic accuracy with optimal cut-offs of 9.09 and 6.88, respectively. Their reported AUCs exceeded 0.90 for both SUV metrics, consistent with our findings, albeit with higher threshold values likely reflecting differences in acquisition parameters or patient populations.

These findings support the integration of quantitative DaTSCAN imaging into multidisciplinary diagnostic workflows. SUV-based thresholds may increase diagnostic confidence in complex or early-stage cases and assist therapeutic decisions, such as balancing dopaminergic versus cholinesterase-inhibitor treatment strategies in mixed DLB/AD presentations [14].

One of the strengths of our study lies in the clinical confirmation of DLB mimics, which included AD, CBD, FTD, MSA, PDD, and vascular dementia. This diagnostic granularity allows the derived SUV thresholds to function not only as markers of scan abnormality but also as predictive indicators of underlying DLB pathology across a broad differential context. Exploratory analyses further suggested that lower striatal SUVmax was associated with visual hallucinations (AUC = 0.70), consistent with prior observations linking dopaminergic loss to this feature in Parkinson’s disease [15]; no significant association was found with REM sleep behaviour disorder, in line with previous PD data [16]. These preliminary findings warrant confirmation in larger cohorts.

Limitations of our study include its retrospective design, modest sample size, and conduct within a single academic center. Quantitative SPECT analysis also requires harmonized acquisition protocols and post-processing tools that may not be widely available, potentially limiting generalizability. An additional methodological limitation is the potential circularity in diagnosis, as DaTSCAN positivity is itself a supportive biomarker in the consensus criteria for DLB. This may introduce bias in the observed association between neuroimaging findings and clinical diagnosis, since the imaging result may have influenced the final diagnostic adjudication in some cases. Furthermore, only 11 of the 33 patients diagnosed with DLB (33%) reported visual hallucinations, which is considerably lower than the reported prevalence of up to 80% for this core clinical feature [6, 17]. This discrepancy likely reflects recall bias, as patients with cognitive decline, memory impairment, or anosognosia may fail to recognize or accurately report these symptoms. Calil et al. reported that 63% of DLB patients with visual hallucinations were unaware of their symptoms, underscoring the role of impaired insight in underreporting [18].

Future studies should explore radiomics approaches to capture spatial heterogeneity of dopaminergic denervation. While such analyses are scarce in DLB, Rahmim et al. demonstrated that radiomic features from DAT SPECT improved prediction of motor decline in Parkinson’s disease [19]. Furthermore, combining striatal quantification with absolute SUV assessment of cardiac sympathetic innervation using I-123 MIBG SPECT may aid differential diagnosis, particularly for PSP and MSA, and provide additional support for the alpha-synuclein origin and connectome (SOC) model of Lewy body diseases [20, 21].

## Conclusion

In conclusion, quantitative I-123-ioflupane SPECT/CT using xSPECT-derived absolute and relative SUV metrics provides an objective and reproducible method for differentiating dementia with Lewy bodies (DLB) from other neurodegenerative conditions with overlapping clinical features. By complementing visual interpretation, SUV-based quantification can enhance diagnostic confidence in uncertain or early-stage cases and contribute to a more standardized, quantitative approach to dopaminergic imaging. Future studies should validate these findings in larger prospective cohorts and explore multimodal or radiomics-based analyses, such as combining striatal SUV metrics with cardiac MIBG imaging, to further refine the diagnostic framework for DLB.

## Supplementary Information

Below is the link to the electronic supplementary material.

Supplementary Material 1 (PNG 731 KB)

## Data Availability

The datasets generated during and/or analysed during the current study are available from the corresponding author on reasonable request.
