# Supplementary figures and images for: Contribution of quantitative I-123-ioflupane SPECT/CT to the differential diagnosis of dementia with lewy bodies
Source: Eur J Nucl Med Mol Imaging. 2025 Nov 27;53(4):2777–85. doi: 10.1007/s00259-025-07680-7 (PMC12920399; doi:10.1007/s00259-025-07680-7)

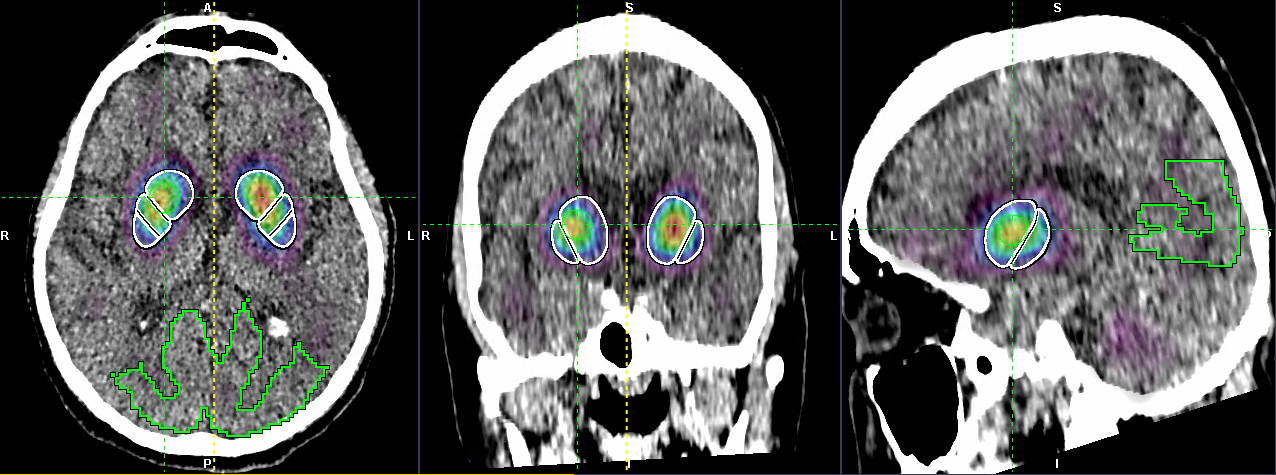

Supplement: Supplementary file 1 — Supplementary Material 1 (PNG 731 KB) [file 259_2025_7680_MOESM1_ESM.png]
